# Physical Activity and Sleep in Chronic Fatigue Syndrome and Fibromyalgia Syndrome: Associations with Symptom Severity in the General Population Cohort LifeLines

**DOI:** 10.1155/2018/5801510

**Published:** 2018-11-04

**Authors:** Monica L. Joustra, Wilma L. Zijlema, Judith G. M. Rosmalen, Karin A. M. Janssens

**Affiliations:** ^1^University of Groningen, University Medical Center Groningen, Department of Psychiatry, Groningen, Netherlands; ^2^University of Groningen, University Medical Center Groningen, Department of Internal Medicine, Groningen, Netherlands

## Abstract

**Objective:**

The aim of the current study was to compare physical activity and sleep duration between patients with chronic fatigue syndrome (CFS), patients with fibromyalgia syndrome (FMS), and controls and to examine the association between physical activity level and sleep duration with symptom severity within these patient groups.

**Methods:**

This study used data of LifeLines, a general population cohort in which 1.0% (*n*=943, 63.7% female, age 44.9 (SD 11.6) years) reported CFS, 3.0% (*n*=2,714; 91.6% female; age 48.4 (SD 10.7) years) reported FMS, and 95.7% (*n*=87,532; 57.9% female; age 44.3 (SD 12.4) years) reported neither CFS nor FMS. Physical activity, sleep duration, and symptom severity were assessed by questionnaires and analysed using ANCOVA and regression analyses, adjusted for age, sex, body mass index, smoking, and educational level.

**Results:**

Patients with CFS and FMS had significantly lower physical activity scores (8834 ± 5967 and 8813 ± 5549 MET ∗ minutes) than controls (9541 ± 5533; *p* < 0.001). Patients with CFS had the longest sleep duration (466 ± 86 minutes) compared to patients with FMS and controls (450 ± 67 and 446 ± 56; *p* < 0.001). A linear association between physical activity, sleep duration, and symptom severity was only found in controls, in whom higher physical total activity scores and longer sleep duration were associated with a lower symptom severity. In contrast, quadratic associations were found in all groups: both relatively low and high physical activity scores and relatively short and long sleep duration were associated with higher symptom severity in CFS, FMS, and controls.

**Conclusion:**

This study indicates that patients with CFS or FMS sleep longer and are less physically active than controls on average. Both low and high levels of physical activity and short and long sleep duration are associated with higher symptom severity, suggesting the importance of patient-tailored treatment.

## 1. Introduction

Functional somatic syndromes (FSS), including chronic fatigue syndrome (CFS) and fibromyalgia syndrome (FMS), are common, disabling, and costly health conditions without known underlying organic pathology [1–4]. CFS is an illness characterised by profound disabling unexplained fatigue [5], while the primary complaint of patients with FMS is unexplained musculoskeletal pain [6]. Both core symptoms are typically accompanied by various additional symptoms. The etiology of CFS and FMS is assumed to be multifactorial including biological, psychological, and social contributing factors [7].

The role of physical activity and sleep in the pathophysiology of CFS and FMS is not well understood. Regarding physical effort, various studies have evaluated the ability of patients with CFS or FMS to perform physical activity, but the results are conflicting [8–10]. There are also different approaches in the way individuals with CFS and FMS cope with physical activity. Recent research suggests that both avoidance of activity and overactivity are associated with an increase in symptom severity, including pain and fatigue [9, 11, 12]. This indicates that, in patients with CFS and FMS, both high and low levels of physical activity may result in higher symptom severity, comparable to what is observed in the general population [13]. Regarding recovery, sleep difficulties have been associated with negative effects on pain and fatigue [14, 15]. A study found that nights with an unusually long or short sleep duration resulted in greater fatigue and that moderate sleep duration was associated with the least fatigue [15]. As with physical activity, an association between sleep duration and symptom severity may thus exist in these patient groups [16].

CFS and FMS are known for substantial clinical and diagnostic overlap. The two conditions are comorbid: 35% to 75% of patients with CFS met the criteria for FMS [17]. This phenomenon resulted in the lumper-splitter discussion [18]. “Lumpers” believe that all FSS result from the same etiology, and “splitters” take the approach that every separate FSS has its own specific background. It is not known to which extent patients with CFS and FMS differ with regard to physical activity and sleep. Studies that compare these associations between patients of one population-based cohort are, to the best of our knowledge, lacking.

The aim of this study was to examine whether patients with CFS, patients with FMS, and controls have different levels of physical activity and sleep duration. Furthermore, we will examine the degree to which physical activity or sleep duration is associated with the severity of physical symptoms, in CFS, FMS, or controls. We hypothesize that both too much and too little physical activity and sleep are related to symptom severity and expect this association to be stronger in patients with CFS and FMS than controls. Furthermore, we hypothesize that CFS is more strongly related to sleep difficulties and FMS is more strongly related to physical activity. These hypotheses were tested within LifeLines, a large population-based cohort study.

## 2. Materials and Methods

### 2.1. The Sample

This study is conducted within the sampling frame of the LifeLines cohort study [19–21], a general population cohort in which 1.0% (*n*=943; 63.7% female; age 44.9 (SD 11.6) years) reported CFS, 3.0% (*n*=2,714; 91.6% female; age 48.4 (SD 10.7) years) reported FMS, and 95.7% (*n*=87,532; 57.9% female; age 44.3 (SD 12.4) years) reported neither CFS nor FMS. The LifeLines cohort study is a multidisciplinary prospective population-based cohort study with a unique three-generation design. LifeLines aims to examine the health and health-related behaviours of more than 167,000 persons living in the North East region of the Netherlands, with a special focus on multimorbidity and complex genetics. It uses a broad range of research procedures to assess biomedical, sociodemographics behavioural, physical, and psychological factors that contribute to health and/or disease of the general population.

#### 2.1.1. Participants

Participants were recruited in two different ways. First, participants aged 25–50 years were invited through a number of general practitioners from the three northern provinces of the Netherlands. Second, persons who were interested to participate in the study could register themselves via the LifeLines website. Patients who agreed to participate were asked to invite their partner, parents, parents-in-law, and children to as well participate in the LifeLines cohort study. Therefore, participants of all age were included in the study. General practitioners evaluated eligibility for participation, whereby persons with severe psychiatric or physical illness, and those not being able to visit the general practitioner and to fill in the LifeLines questionnaires, and/or persons those who did not understand the Dutch language were excluded from the study. However, children and parents were not excluded in the case of the mentioned exclusion criteria, when a representative was willing to assist these persons in the performance of the study. In case of pregnancy, participation was rescheduled until 6 months after pregnancy or 3 months after breastfeeding.

The LifeLines cohort study obtained approval by the Medical Ethical Committee of the University Medical Center Groningen. All participants received written information on the purpose and methods of the LifeLines cohort study. Written informed consent of participants was obtained after the procedure of the LifeLines cohort study was fully explained. Data of the LifeLines cohort study are kept confidential and are only used for medical research.

### 2.2. Measures

#### 2.2.1. Chronic Fatigue Syndrome and Fibromyalgia Syndrome

CFS and FMS were assessed by means of a self-report questionnaire, including a list of chronic disorders including CFS and FMS. The participants were asked to indicate which of these disorders they had or have had. More than one answer to this question was allowed. Participants who reported both CFS and FMS were excluded (*n*=264), since we were interested in differences between both conditions. Controls were defined by the absence of CFS and FMS.

#### 2.2.2. Physical Activity and Sleep Duration

Physical activity was assessed by means of the validated Short Questionnaire to Assess Health-enhancing physical activity (SQUASH) [22]. This self-report questionnaire assesses physical activity undertaken in an average week in the past months across a set of domains. These domains include commuting activities (walking or bicycling to/from work or school), leisure-time activities (walking, bicycling, gardening, and odd jobs), sports activities, household activities, and activities at work and school. It is a reliable and valid questionnaire [22]. The SQUASH discusses three questions per activity: days per week of the activity (frequency), average time per day (duration in minutes), and intensity of the activity. The intensity of the physical activity was scored on a 3-point scale ranging between (1) “*Slow*,” (2) “*Moderate*,” and (3) “*Fast*.”

The answers collected with the SQUASH can be examined as a continuous measure by weighting each type of activity by its energy requirements defined in intensity scores, also referred to as metabolic equivalent of tasks (METs). METs are defined as multiples of the resting metabolic rate, thus the energy expenditure at rest. Selected MET values are derived using the Ainsworth's Compendium of Physical Activities [23]. Based on age and assigned MET values, physical activities were subdivided into three intensity categories: light, moderate, and vigorous. For adults aged 18–54 years, the following cutoff values were used: <4.0 MET (light intensity), 4.0 to 6.5 MET (moderate intensity), and ≥6.5 MET (vigorous intensity), and for adults aged ≥55 years, these cutoff values were <3.0 MET (light), 3.0 to 5.0 MET (moderate), and ≥5.0 MET (vigorous). The three MET categories were combined with self-reported intensity for each activity, resulting in a combined intensity score ranging from 1 to 9, with 1 being light MET and light self-reported intensity and 9 being vigorous MET and vigorous self-reported intensity. The classification of physical activities according to the combined intensity score was <3 (light intensity), 3 to 6 (moderate intensity), and ≥6 (vigorous intensity). The physical activity scores of the different domains were calculated by multiplying duration (minutes per week) with the MET value, taking into account the combined intensity score. Subjects with unlikely values were excluded if separate activity categories exceeded plausible values, more than two activity categories of the questionnaire were missing, and/or ≥18 hours/day were spent on all activities together.

Sleep duration was assessed using the question: “How many minutes do you sleep on average per day?”

#### 2.2.3. Symptom Severity

Symptom severity was assessed with the 12-item somatization scale of the Symptom CheckList-90 (SCL-90 SOM) [24]. The SOM scale measures self-reported intensity of somatic symptoms. This scale consists of 12 somatic symptoms, including a lump in your throat, faintness or dizziness, feeling weak in parts of your body, headaches, heavy feelings in arms or legs, hot or cold spells, nausea or upset stomach, numbness or tingling in your body, pains in heart or chest, pains in lower back, soreness of your muscles, and trouble getting your breath. Participants were asked to what extent they have been limited by these somatic symptoms in the past seven days. The somatic symptoms were scored on a 5-point scale ranging from (1) “*Not at all*” to (5) “*Extremely*.” An additional item assessing fatigue was used from the RAND-36 [25]: “How much of the time during the past four weeks did you feel tired?” This item was scored on a six-point scale ranging from (1) “*All of the time*” to (6) “*None of the time*.” The fatigue score was transformed to a 5-point scale with (1) “*None of the time*” to (5) “*All of the time*,” with a combined score of (3) “*A good bit of the time*” and (4) “*Some of the time*” into (3) “*quite a bit*” to obtain consistency with the SOM scale. Symptom severity was calculated by taking the mean score of the 13 somatic symptoms. Therefore, the total symptom severity ranged from (1) all symptoms endorsed as “*Not at all or none of the time*” to (5) all symptoms endorsed as “*Extremely or all of the time*.”

#### 2.2.4. Covariates

Length in centimetres and weight in kilograms were assessed during a basic medical examination at a local LifeLines research facility. Subsequently, body mass index (BMI) was calculated as kg/m^2^. The smoking status was assessed using the following question: “Do you smoke now, or have you smoked in the past month?” Participants could fill in “*yes*” or “*no*.” Educational level was assessed using the following question: “What is your highest completed education?,” resulting in information about low, middle, and high educational level. Low educational level was defined as lower secondary education or less, middle educational level was defined as higher secondary education, and high educational level was defined as tertiary education.

### 2.3. Statistical Analyses

For all continuous variables, means ± standard deviations (SDs) were calculated. One-way analyses of variance (ANOVA) were performed for continuous data, to test the differences in sample characteristics. Differences in symptom severity were also investigated between males and females within the different study groups. In addition, *χ*^2^ tests were performed for categorical data. For continuous variables, analyses of covariance (ANCOVA) with post hoc Bonferroni correction were performed to examine differences in physical activity level and sleep duration between patients with CFS, patients with FMS, and controls. In addition, sex differences in physical activity and sleep duration were explored. Linear and quadratic regression analyses were conducted using standardized variables to examine how physical activity and sleep duration were associated with symptom severity in the different groups. Four regression models were performed: both linear and regression analyses for physical activity and for sleep duration. All analyses were adjusted for age, sex, BMI, smoking status, and educational level, since they are known to be related to CFS [26, 27], FMS [28–30], physical activity [31, 32], and sleep [33, 34]. All analyses were performed using SPSS version 20. Statistical significance was defined as *p* < 0.05.

## 3. Results

### 3.1. Sample Characteristics

Data were available for 91,453 participants; descriptives, including age, BMI, education, SOM score, sex, and smoking, are shown in Table 1. Of these participants, 1.0% (*n*=943) reported CFS, 3.0% (*n*=2,714) reported FMS, and 95.7% (*n*=87,532) reported neither CFS nor FMS. Women were most prevalent in all groups. The mean age varied between 44.3 ± 12.4 for controls, 44.9 ± 11.9 for patients with CFS, and 48.4 ± 10.7 years for patients with FMS. Female CFS patients and controls reported significantly higher symptom severity (2.1 ± 0.6 and 1.5 ± 0.4, respectively) compared to males (1.9 ± 0.6 and 1.4 ± 0.3), while no difference in symptom severity was found in female FMS patients (2.0 ± 0.5) compared to male FMS patients (1.9 ± 0.5).

### 3.2. Physical Activity and Sleep Duration

**Figure 1:**
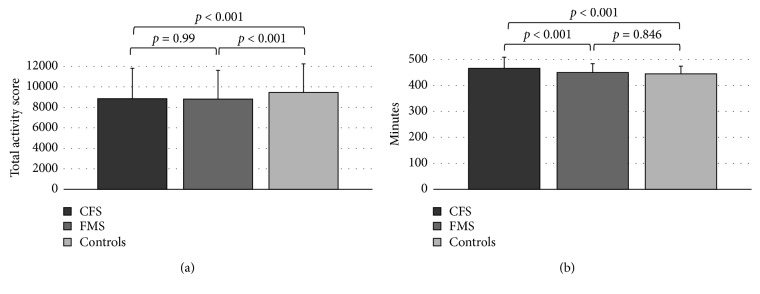
(a) Physical activity. (b) Sleep duration. CFS = chronic fatigue syndrome; FMS = fibromyalgia syndrome. Analyses of covariance and Bonferroni correction were done, adjusted for age, sex, BMI, smoking, and education.

Physical activity levels in patients with CFS, patients with FMS, and controls are shown in Figure 1(a). ANCOVA analysis revealed significant groups' differences (*F*(7,76182)=303, *p* < 0.001). Post hoc comparisons with Bonferroni correction indicated that patients with CFS and FMS had a significantly lower physical total activity score than controls (8834 ± 5967 and 8813 ± 5549 MET ∗ minutes, respectively, versus 9541 ± 5533; both *p* < 0.001). There was no significant difference in physical total activity score between patients with CFS and FMS (*p*=0.99). Lastly, males were significantly more physically active than females among all three study groups.

Sleep duration in patients with CFS, patients with FMS, and controls is shown in Figure 1(b). ANCOVA analysis revealed significant groups' differences (*F*(7,39438)=222, *p* ≤ 0.001). Post hoc comparisons with Bonferroni correction indicated that patients with CFS had the longest sleep duration (466 ± 86 minutes) compared to patients with FMS and controls (450 ± 67 and 446 ± 56, respectively, both *p* < 0.001), while no difference was found between patients with FMS and controls (*p*=0.846). Furthermore, female CFS patients and controls reported significantly longer sleep duration (474 ± 84 and 453 ± 59 minutes, respectively) than males in the corresponding groups (453 ± 87 and 437 ± 50 minutes), while no difference in sleep duration was found between female FMS patients (451 ± 66 minutes) and male FMS patients (442 ± 80 minutes).

### 3.3. Physical Activity or Sleep Duration Associated with Symptom Severity

Physical activity and sleep duration showed both linear and quadratic associations with symptom severity. Results of both linear and quadratic regression analyses are shown in Table 2. Linear regression analyses showed that, only in controls, physical total activity score (model 1) and sleep duration (model 2) were related to symptom severity; controls with a higher physical total activity score or longer sleep duration reported a slightly lower symptom severity. No significant linear associations were found in patients with CFS or FMS between physical total activity score or sleep duration and symptom severity.

Quadratic regression analyses indicated a significant association between total physical activity score in CFS, FMS, and controls (model 3). Both linear and quadratic terms were significant in FMS and controls, and only the quadratic term, but not the linear, was significant in CFS. Thus, patients with CFS, patients with FMS, and controls with relatively low and high physical activity scores reported higher symptom severity than those with moderate physical activity scores. Furthermore, all three groups showed significant quadratic associations between sleep duration and symptom severity (model 4). Both linear and quadratic sleep terms were significant in patients with CFS and controls, while only the quadratic but not the linear sleep term was significant in patients with FMS. Thus, patients with CFS, patients with FMS, and controls with short or long sleep duration reported a higher symptom severity than those with moderate sleep duration.

## 4. Discussion

This study revealed that patients with CFS and FMS were significantly less physically active than controls. Furthermore, patients with CFS reported longer sleep duration than patients with FMS and controls. Only in controls, physical total activity score and sleep duration were linearly related to symptom severity, with both higher physical total activity score and higher sleep duration being associated with slightly lower symptom severity. Quadratic associations were present in all groups; both relatively high and low physical activity levels were associated with higher symptom severity in patients with CFS, patients with FMS, and controls; and both relatively long and short sleep duration were associated with higher symptom severity in patients with CFS, patients with FMS, and controls.

The main strength of this study is the large population cohort. To the best of our knowledge, this is the first study that evaluates physical activity and sleep duration in patients with CFS and FMS in one large population cohort. A sufficient number of patients with CFS and FMS were identified, allowing for meaningful statistical comparisons. Moreover, the large number of patients enabled examining the association between sleep duration, physical activity, and symptom severity in CFS, FMS, and controls. Both patients and controls with different physical activity or sleep duration outcomes were therefore present in the cohort. Finally, since LifeLines is a large cohort study with extensive measurements, adjusting for important covariates such as age, sex, BMI, smoking status, and educational level was possible.

Our study also contained limitations, including the use of a self-report questionnaire for the assessment of CFS and FMS. Instead of current diagnoses, our questionnaire asked for a history of CFS and FMS. A previous study in a general population cohort from the same geographical area indicated that about 75% and 100% of the participants that reported a history of CFS and FMS, respectively, still had this syndrome at the time of reporting [35]. In addition, self-reports may underestimate the amount of persons with FSS. This seems not likely in our study because the prevalence rates for CFS and FMS were comparable to previous studies [27, 29]. Moreover, the majority of the patients with CFS and FMS in the current study recently experienced fatigue and musculoskeletal pain. Furthermore, subjective measurements were used to assess sleep duration and physical activity, instead of objective measures. For example, sleep duration was assessed using a single question, so participants may have interpreted this differently (e.g., time in bed, actual time sleep, and inclusion of naps). A final limitation is that the cross-sectional design did not allow conclusions on cause-and-effect relationships.

In line with previous findings, this study revealed that both patients with CFS and FMS were significantly less physically active than controls [36, 37]. However, it should be mentioned that self-reported questionnaires to assess physical activity levels in these patient groups have shown a low reliability [38, 39]. In contrast to our hypothesis, no difference in physical activity was found between patients with CFS and FMS. Lower activity levels in patients with CFS or FMS might be explained by the substantial limitations in physical functioning that may be caused by their symptoms [9]. In addition, a lack of physical activity might also contribute to physical deconditioning, further increasing symptom severity [12, 40]. We found that both low and high physical activity levels in patients with CFS, patients with FMS, and controls were associated with the reporting of more symptoms. This finding stresses the close relationship between physical activity and the experience of symptoms. Thus, on the one hand, low activity levels may be associated with the experience of more and more severe symptoms, while on the other hand, high physical activity level may exacerbate symptoms in CFS and FMS [9, 11, 12].

Differences between patients with CFS and FMS were found for sleep duration, since patients with CFS were found to report longer sleep duration than patients with FMS and controls. However, misestimation of sleep duration appears common in patients with CFS and FMS, particularly in patients having a poor sleep quality [41, 42]. Nevertheless, our results are in accordance with our hypothesis and might be due to the primary complaint of disabling fatigue in patients with CFS [5, 43, 44]. Furthermore, our results are in line with a recent study that reported that nights with an unusually long or short sleep duration resulted in greater fatigue, and that moderate sleep duration was associated with the least fatigue [15].

Our study also revealed differences between patients, as illustrated by the finding of quadratic associations of symptom severity with physical activity and sleep. These quadratic associations indicate that the pathophysiological role of physical activity and sleep varies not only between but also within patient groups with CFS or FMS. Treatment aimed at reducing symptoms might therefore better be tailored to individual patients. This is mainly important since both CFS and FMS are characterised both by nonrestorative sleep and intolerance to physical exercise. Since the LifeLines cohort is a large population cohort study that aims to study a wide spectrum of mental and somatic disorders, it was not feasible to more extensively assess lifestyle factors such as physical activity and sleep in CFS and FMS during the baseline assessment because of practical limitations. We aim to include objectively measured lifestyle factors in CFS and FMS in future assessment waves. Further studies will be necessary to determine the effect of objectively measured physical activity or sleep duration, by using, for instance, polysomnography or accelerometers. Furthermore, the association between sleep duration and symptom severity was found to vary between different patients. Therefore, studies that evaluate how sleep duration and physical activity are related to symptom severity within individual patients, so called idiographic research [45], is recommended to further study the role of sleep and physical activity in patients with CFS and FMS.

## 5. Conclusion

This study revealed that, on average, patients with CFS and FMS sleep longer and are less physically active than controls and that both high and low levels of physical activity and sleep duration are associated with higher symptom severity. Differences were found within patient groups, suggesting etiological heterogeneity in these patients and thus the importance of patient-tailored treatment.

## Figures and Tables

**Table 1:** Sample characteristics.

				Pairwise comparisons^c^; *p* value		
	CFS	FMS	Controls	CFS vs FMS	CFS vs controls	FMS vs controls
*Mean (SD)*						
Number (%)	943 (1.0)	2714 (3.0)	87532 (95.7)			
Age^a^	44.9 (11.6)	48.4 (10.7)	44.3 (12.4)	<0.001	0.137	<0.001
BMI (kg/m^2^)^a^	26.4 (4.8)	27.8 (5.3)	26.0 (4.3)	<0.001	0.407	<0.001
Symptom severity (1–5)^a^	2.0 (0.6)	2.0 (0.5)	1.5 (0.4)	0.038	<0.001	<0.001

*n(%)*						
Education^b^						
Low	319 (33.8)	1193 (44.0)	25,418 (29.0)	<0.001	<0.001	<0.001
Middle	377 (40.0)	1055 (38.9)	34,211 (39.1)			
High	213 (22.6)	377 (13.9)	25,697 (29.7)			
Female^b^	601 (63.7)	2485 (91.6)	50,705 (57.9)	<0.001	<0.001	<0.001
Smoking^b^	257 (27.3)	609 (22.4)	18,520 (21.2)	0.002	<0.001	0.145

CFS = chronic fatigue syndrome; FMS = fibromyalgia syndrome. ^a^ANOVA; ^b^*χ*^2^ test; ^c^Bonferroni correction for continuous and *χ*^2^ test for categorical variables.

**Table 2:** Associations between physical activity, sleep duration, and symptom severity.

	CFS		FMS		Controls	
	*B*	95% CI	*B*	95% CI	*B*	95% CI
*Linear*						
(1) Physical total activity score	−0.007	−0.047, 0.032	−0.009	−0.031, 0.012	−0.007^*∗∗*^	−0.009, −0.004
(2) Sleep duration	−0.005	−0.045, 0.036	−0.009	−0.036, 0.017	−0.004^*∗*^	−0.008, −0.001

*Quadratic*						
(3) Physical total activity score (linear term)	−0.042	−0.091, 0.008	−0.046^*∗∗*^	−0.073, −0.020	−0.009^*∗∗*^	−0.012, −0.006
Physical total activity score (quadratic term)	0.020^*∗*^	0.003, 0.038	0.019^*∗∗*^	0.011, 0.027	0.001^*∗∗*^	0.001, 0.002
(4) Sleep duration (linear term)	−0.045^*∗*^	−0.089, 0.00	−0.021	−0.047, 0.005	−0.007^*∗∗*^	−0.011, −0.003
Sleep duration (quadratic term)	0.017^*∗∗*^	0.008, 0.025	0.040^*∗∗*^	0.031, 0.049	0.00^*∗∗*^	0.00, 0.00

CFS = chronic fatigue syndrome; FMS = fibromyalgia syndrome. ^*∗*^*p* ≤ 0.05; ^*∗∗*^*p* ≤ 0.001. Multivariable regression analyses were done, adjusted for age, sex, BMI, smoking, and education.

## Data Availability

The LifeLines data used to support the findings of this study were supplied by the LifeLines cohort study under license and so cannot be made freely available. Requests for access to these data can be made at https://www.lifelines.nl/.

## Abbreviations

FSS: Functional somatic syndromes
CFS: Chronic fatigue syndrome
FMS: Fibromyalgia syndrome.
